# Addressing the intersection between alcohol consumption and antiretroviral treatment: needs assessment and design of interventions for primary healthcare workers, the Western Cape, South Africa

**DOI:** 10.1186/s12992-016-0201-9

**Published:** 2016-10-26

**Authors:** M. Schneider, M. Chersich, M. Temmerman, C.D. Parry

**Affiliations:** 1 Alcohol, Tobacco and Other Drug Research Unit (ATODRU), Medical Research Council, P O Box 19070, Tygerberg 7505, Cape Town, South Africa; 2 Wits Reproductive Health and HIV Institute, Faculty of Health Sciences, University of Witwatersrand, Johannesburg, South Africa; 3 Department of Obstetrics and Gynaecology, International Centre for Reproductive Health (ICHR), Ghent University, Ghent, Belgium; 4 Department of Psychiatry, Stellenbosch University, Stellenbosch, South Africa

**Keywords:** Antiretroviral treatment, Alcohol, Primary healthcare, South Africa

## Abstract

**Background:**

At the points where an infectious disease and risk factors for poor health intersect, while health problems may be compounded, there is also an opportunity to provide health services. Where human immunodeficiency virus (HIV) infection and alcohol consumption intersect include infection with HIV, onward transmission of HIV, impact on HIV and acquired immunodeficiency syndrome (AIDS) disease progression, and premature death. The levels of knowledge and attitudes relating to the health and treatment outcomes of HIV and AIDS and the concurrent consumption of alcohol need to be determined.

This study aimed to ascertain the knowledge, attitudes and practices of primary healthcare workers concerning the concurrent consumption of alcohol of clinic attendees who are prescribed antiretroviral drugs. An assessment of the exchange of information on the subject between clinic attendees and primary healthcare providers forms an important aspect of the research. A further objective of this study is an assessment of the level of alcohol consumption of people living with HIV and AIDS attending public health facilities in the Western Cape Province in South Africa, to which end, the study reviewed health workers’ perceptions of the problem’s extent. A final objective is to contribute to the development of evidence-based guidelines for AIDS patients who consume alcohol when on ARVs. The overall study purpose is to optimise antiretroviral health outcomes for all people living with HIV and AIDS, but with specific reference to the clinic attendees studied in this research.

**Methods:**

Overall the research study utilised mixed methods. Three group-specific questionnaires were administered between September 2013 and May 2014. The resulting qualitative data presented here supplements the results of the quantitative data questionnaires for HIV and AIDS clinic attendees, which have been analysed and written up separately. This arm of the research study comprised two, separate, semi-structured sets of interviews: one face-to-face with healthcare workers at the same primary healthcare clinics from which the clinic attendees were sampled, and the other with administrators from the local government health service via email. The qualitative analysis from the primary healthcare worker interviews has been analysed using thematic content analysis.

**Results:**

The key capacity gaps for nurses include the definition of different patterns and volumes of alcohol consumption, resultant health outcomes and how to answer patient questions on alcohol consumption while on antiretroviral treatment. Not only did the counsellors lack knowledge regarding alcohol abuse and its treatment, but they were also they were unclear on their role and rights in relation to their patients. Doctors highlighted the need for additional training for clinicians in diagnosing alcohol use disorders and information on the pharmacological interventions to treat alcoholism.

**Conclusion:**

Pertinent knowledge regarding patient alcohol consumption while taking ARVs needs to be disseminated to primary healthcare workers.

## Background

Research indicates that worldwide, South Africa has the highest number of new HIV infections, the largest public sector antiretroviral treatment (ART) programme [37], and one of the highest levels of alcohol consumption per drinker [40].

Alcohol consumption likely plays a key but under-acknowledged role in HIV disease progression and survival [13]. The interface between alcohol consumption and HIV and AIDS has been largely overlooked in public health research [18]. A recent review of research into alcohol and HIV in Sub-Saharan Africa highlights the lack of research on the association between alcohol use and HIV disease progression [31]. Alcohol consumption is common, however, among those with HIV infection, with usage ranging from 37 to 68 % and hazardous usage from 5 to 28 % in selected populations [14]. Results from a meta-analytic review of published studies examining the association of alcohol use and ART adherence, support a significant and reliable association of alcohol use and medication non-adherence [21]. Studies have demonstrated a temporal and dose-response relationship between alcohol consumption and non-adherence to ARVs, with non-binge drinkers missing more doses than non-drinkers, and binge drinkers missing more doses than non-binge drinkers [6]. Furthermore, binge drinking rather than the cumulative exposure of hazardous drinkers was predictive of non-adherence to ART [7]. A recent study conducted in HIV clinics in Tshwane, Gauteng province in South Africa, found a significant association between level of alcohol use and degree of non-adherence [27].

South Africa’s average alcohol consumption in 2010 was 10 % above the global average at 27.1 l of pure alcohol per annum [40]. In South Africa in 2010, the prevalence of alcohol use disorders (AUDs) and alcohol dependence were respectively 10 and 4 % in men and 1.5 and 0.7 % in women. These figures are three times higher than the average for the whole of Africa [40]. Findings by Azar et al. [2] support an association between AUDs and decreased adherence to antiretroviral therapy, and poor HIV treatment outcomes among HIV-infected individuals.

The increased life expectancy for HIV-infected individuals made possible by ART increases the cumulative competing risk of long-term adverse outcomes associated with alcohol consumption [14]. Furthermore, the wider availability of ART may lead to an increase in risky behaviour. Shisana et al. [37] stress that this risk, termed “risk compensation”, needs to be reduced.

An American study [36] examined the influence of patient beliefs about alcohol on ART adherence and elucidated clinician beliefs about drinking and taking ART. Most patients (85 %) believed alcohol and ART should not be mixed. Alcohol was found to affect adherence through a decision to skip ART doses when drinking rather than through drunken forgetfulness. In addition, more than 50 % of clinicians believed alcohol and ART should not be taken together. These findings have implications for patient care and training for clinicians in this domain. Given the scaling up in recent years of the ARV roll-out in the South African public health sector [12] in response to the twin goals of high ARV coverage and optimal ARV treatment outcomes, ineffective antiretroviral therapy management, particularly at primary healthcare level, would result in adverse health outcomes and wastage of scarce health resources.

In South Africa, primary healthcare workers (PHCWs) in ART clinics are responsible for life-long treatment and support to avert exacerbation of the existing health condition. The PHCWs also play a prevention role, specifically for the onward transmission of HIV. In terms of ART management options, Callaghan et al.’s [10] literature review found task shifting to be a feasible means of addressing human resource shortfalls in HIV care in sub-Saharan Africa. Task-shifting in this context involves shifting some health services to less qualified personnel.

Bearing in mind the under-resourced public health services, high HIV prevalence and an expanded ART programme utilising nurse-initiated management of antiretroviral treatment, a research study was conducted in the public health sector in the Western Cape Metro region of the Western Cape, a province in South Africa. The eight sample ART clinics were located in, or drew patients from under-privileged communities, which means the patient pool can be considered fairly homogenous in terms of the social determinants of health that have an impact on alcohol misuse, HIV infection and access to health services.

The study aims to gauge knowledge levels and understanding of both ART clinic attendees and the PHCWs of the implications of alcohol consumption while taking ARVs. This paper focuses on the PHCWs (medical doctors, nurses, ART counsellors). We ascertained the PHCWs understanding of the interface between alcohol and HIV and the kinds of information they were imparting to healthcare service users on the adverse effects of alcohol use and the adverse effects of alcohol consumption on the course of the disease.

A further objective of this study is to present PHCWs’ assessment of the level of alcohol consumption of people living with HIV and AIDS (PLHA) who attend public health facilities in the Western Cape Province in South Africa. Given the apparent absence of an international best practice standard, or national, provincial or other guidelines on alcohol consumption and ART, the study endeavours to establish whether there are any internal clinic guidelines in this respect, and in addition, PHCW and health service administrator views on the availability of such guidelines. The overarching study purpose is to optimise ART outcomes, contributing to the development of evidence-based guidelines for AIDS patients who consume alcohol while on ARVs is also an important objective in this regard.

## Methods

### Design

This study employed qualitative research methods and was cross-sectional in nature.

### Sampling

The study involved eight sets of three PHCWs, and two health service administrators at local government level. Data was collected by key informant interviews with PHCWs (medical doctors, nurses, ART counsellors) at primary healthcare clinics located in Western Cape Metro region, which comprises eight sub-districts: Eastern, Khayelitsha, Klipfontein, Mitchell’s Plain, Northern, Southern, Tygerberg and Western. The two key informants at local government level were interviewed using a separate semi-structured questionnaire.

Three staff members, an ART counsellor, a doctor and a nurse, were interviewed to determine the level of healthcare worker knowledge at primary level clinics dispensing ART in the public health sector regarding the effect of alcohol consumption combined with antiretroviral drugs. The facility manager allocated the PHCWs to be interviewed, making recruitment for each cohort by convenience sampling. Interviews were conducted with three PHCWs, one from each cohort, in each of the eight selected clinics, that is, 24 interviews with PHCWs in total.

City Health manages the clinics and the Provincial department of health is responsible for hospitals and most of the community health centres. Interviews were conducted with a designated administrative staff member responsible for implementing the South African roll-out of ART programmes in the City of Cape Town and the Western Cape Government, respectively. These interviews ascertained policy directives and guidelines on alcohol consumption while on ART.

### Study instruments

The questions on the PHCW and health service administrator questionnaires were open-ended, with each question having a series of prompts for the respondents. Besides determining knowledge and understanding in this research area, the semi-structured questionnaire (for PHCWs) probed attitudes and practices relating to alcohol consumption and ART, as well as the difficulties PHCWs experience pertaining to ARV regimen adherence for clinic attendees.

### Study procedures

Over the course of a week, an experienced research project manager gave fieldworkers training that included training workshops on interviewing skills and techniques, and lecture on research ethics. The latter covered the process of obtaining informed consent from participants and emphasising the confidentiality of the questionnaire content with assurances that answers could not be linked to individual respondents.

Interviewees gave written, informed consent and granted permission to have the interview recorded. In addition, notes were taken, capturing the responses to the semi-structured interviews as they were conducted. For quality control, the project manager sat in on six of the interviews for which the interviewees granted permission. Interviewees made an informed and voluntary decision about accepting or declining participation in the study.

Ethics approval for the research was obtained from the University of Stellenbosch, (Ethics reference number: N11/07/223), and the research protocol was edited to match the design and analysis requirements of the funding facilitators, namely, the United States Centers of Disease Control and Prevention.

In addition to comparing the data from the three PHCW groups from the eight clinics in the study, data was also compared from each of the three groups of healthcare workers at one randomly selected clinic out of the eight sample clinics.

The research element involving the health service administrators necessitated contacting the City of Cape Town and Western Cape Province offices to ascertain the details of a spokesperson with knowledge of the working of ART programmes from each office. These individuals were contacted by telephone to introduce the research project. After agreeing to participate, they completed the questionnaires and emailed them to the principal investigator (MS). These administrative officers completed a separate, semi-structured questionnaire to the one conducted with the PHCWs. This data collection occurred in parallel to the PHCW data collection.

### Examples of questionnaire questions

The investigation focused firstly on whether the PHCWs perceive alcohol consumption as a problem among the clinic population and the community from which the clinic draws its patients. A further question posed concerned PHCWs’ perceptions of the nature of the patient-service provider relationship, and in particular how the PHCWs view their role in relation to patients who abuse alcohol. The extent to which taking ARVs and drinking alcohol occurred simultaneously was explored by asking, for example, whether participants believed that clinic attendees intentionally skip ARV doses when drinking or intending to drink.

Health service providers were also asked whether there were clear guidelines for patients concerning the concurrent use of alcohol and ARVs. The questionnaires ascertained PHCW views on unmet needs relating to training and information dissemination on the role of alcohol consumption and the course of HIV and AIDS. Interviews were conducted with the health service administrators to determine official health service policy guidelines in this domain. To this end, this group’s questionnaire probed current policy directives and guidelines on alcohol consumption while on ART.

### Data analysis

All 24 PHCW interview recordings were transcribed and subjected to thematic content analysis [17]. The transcripts were then checked against specific knowledge items relating to alcohol abuse and its treatment, and whether the PHCWs knew what constituted sensible or non-risky drinking for PLHA. The specific knowledge items were found in the Diagnostic and Statistical Manual of Mental Disorders-IV, (DSM-IV), [1] which defines the alcohol use disorders, namely, alcohol dependence and alcohol abuse. Other knowledge items were found in the Alcohol Use Disorders Identification test (AUDIT) [9], which is used to assess patients’ alcohol use and timeously detect hazardous and harmful alcohol use in primary healthcare settings.

The principal investigator analysed all the transcripts and an independent researcher in qualitative research analysed a sample of randomly drawn transcripts from the three batches. The themes emerged as a result of reading and comparing the respondent opinions in the transcripts and determining the most common responses. Illustrative quotes pertaining to these themes are provided in the text.

Coherence in terms of knowledge, information disseminated and a common policy agenda within a particular clinic was also examined as well as how the different PHCWs within the sample clinics viewed each other’s roles in this area. This aimed to ascertain whether there were within-clinic differences among PHCWs and to compare these to the findings across the different clinics.

The principal investigator and the project manager reached consensus by independently comparing the completed questionnaires from the two health service administrators and discussing the findings.

## Results

### Main themes emerging from the PHCW interviews

PHCWs had different types of expertise and various levels of and training and experience at different levels across clinics. The emerging themes within the different groups point towards specific interventions being required for each level of PHCW to improve service in the primary healthcare clinics.

### Interviews with doctors

The doctors emphasised to patients that reactions between alcohol and ARVs make the ARVs less effective. They also stressed the negative effect of alcohol on the liver and that a damaged liver cannot process the ARVs. Patients with tuberculosis or hepatitis were told to avoid alcohol completely.

Besides the clinical effects of alcohol use on ART, the most important outcome for the physicians was the impact of alcohol use on ART compliance. For the clinicians, the main issue with alcohol consumption, heavy drinking in particular, was that patients defaulted on treatment. More than one doctor mentioned having to be careful about telling patients not to mix alcohol and ARVs as alcohol would be chosen above the ARVs. Patients were, however, generally told to take their medication irrespective of whether they were drinking or intending to drink alcohol. One doctor went so far as to advise patients to “take their ARVs with alcohol” rather than stop treatment; another reported that community care workers tracking defaulters find them in “shebeens” or taverns.

An important aspect of the clinicians’ knowledge in this domain is expertise in screening for alcohol problems, diagnosis of AUDs and required interventions for problematic alcohol use. It is also important to ascertain whether clinic attendees know the definition of harmful drinking. The doctors were familiar with the Cutting down, Annoyance by criticism, Guilty feeling and Eye-openers (CAGE)^1^ questionnaire, but less so with the Alcohol Use Disorders Identification Test (AUDIT). They were aware of organisations for substance abuse treatment, such as the South African National Council on Alcoholism and Drug Dependence (SANCA). Those interviewed did not know about Brief Interventions (BIs) to treat alcohol problems, with the exception of one doctor, they were generally not acquainted with the term “binge drinking”^2^ and they did not know of policy guidelines relating to ART and concomitant alcohol consumption.

Some of the clinicians were unsure of the clinical standards used for diagnosing AUDs; furthermore, they did not mention either the World Health Organization, International Classification of Disease-10 ICD-10, [39], or American Psychiatric Association: Diagnostic and Statistical Manual of Mental Disorders-IV, (DSM-IV), [1] classification systems. They also had no detailed knowledge of pharmacological interventions to treat alcoholism. One doctor said he would welcome additional training on substance abuse and ARVs and another suggested that once screened and identified as having an alcohol problem, the patient should be referred to a rehabilitation centre as given the high patient volume and workload they would be unable to provide adequate follow-up.

Different reasons were given for the heavy workload by the different healthcare practitioners. Generally, it was not the number of patients per se, but the nature of the presenting medical problems that were identified as time consuming. One doctor mentioned that advanced, complicated or adherence-lapsed cases took longer, and that in addition, when administrative systems were not sufficiently effective to allow quick access to patient records, delays were inevitable.

Besides substance abuse, the doctors mentioned several other factors that resulted in non-adherence to ART, including “pill fatigue”, depression and non-disclosure of their HIV status. It was mentioned that non-adherence might be indirectly related to alcohol consumption, either the patient’s own or that of other family members. A patient with an abusive partner abusing alcohol may seek shelter elsewhere, away from home and the clinic, and as a result default on ART.

The doctors’ comments about the ART counsellors included the following: “lack basic knowledge of HIV and AIDS” and “should be better trained”. One respondent recommended that the counsellor’s role should be extended beyond the initiation of ART and the follow-up of defaulters to include counselling for substance abuse. Another stated that the counsellors “can benefit from more training on substance abuse”. Doctors found that some clinic attendees were illiterate and in general did not appear to have community support.

### Interviews with nurses

The nurses were familiar with the CAGE assessment for alcohol abuse [15], while a few also knew about the AUDIT questionnaire [9]. They were not aware of policy guidelines with regards to alcohol and ART and had no knowledge of BIs. A few were familiar with SANCA but they were not well-informed about medication for alcoholism and most of them could not define “binge drinking”. The nurses would appreciate training to deal with substance abuse for HIV-positive patients and also believe that the counsellors’ role should be extended to focus on substance abuse.

They understood that certain ARVs are contraindicated for alcohol, for example, one nurse stated that Efavirenz cannot be taken with alcohol as it is hallucinogenic, and that Nevirapine and alcohol both have a negative impact on the liver. Gauging from their responses to the questionnaire, the nurses recognised a worsening of co-morbid conditions such as TB, epilepsy, diabetes and other cardio-vascular diseases when HIV patients drink excessively.

Some of the respondents felt that because of the client-service provider relationship, they were in a position to address alcohol problems without being judgmental. Others felt that the opposite was true, that is broaching the subject of alcohol consumption may harm the patient-service provider relationship: “People should be allowed to live their lives”. One respondent maintained that “there is not much we can do for patients who consume alcohol–this is the way they cope in their community”. One interviewee even maintained that those who drink and take their ARVs have suppressed viral loads.

Usually the defaulters were patients with alcohol problems while some of the patients defaulted due to logistical problems with access to ARVs. Non-adherence was also linked to the stigma related to HIV as some patients had not disclosed their status to co-habitees and therefore did not want to be seen taking their ARVs. Nurses mentioned that when first-line treatment failed, a number of patients had needed to go on second- and even third-line treatment for AIDS, the latter being more complex with more side-effects. In general, the nurses stated that patients were often in denial concerning their HIV status and tended to be poorly informed and lacking in knowledge of matters pertaining to their health and well-being.

### Interviews with counsellors

The counsellors emphasised to the patients that ARVs are life-long medication: “ARVs is not a Panado, just for now”. They maintain that while alcohol consumption often results in missed ARV doses, the person could also default on treatment, i.e. stop taking ARVs for an extended period of time. The counsellors mentioned that besides passing out from excessive alcohol consumption and missing ARV doses, the patients did not always remember whether they had taken their ARVs and therefore overdosed. Clinic appointments are pre-booked and patients may miss or arrive late for appointments if drunk. Non-attendance at the clinic as scheduled is an aspect of non-adherence for ART that is monitored.

Counsellors were generally not knowledgeable about BIs or the AUDIT, while some were familiar with the CAGE or medication for addiction and the term “binge drinking”, and commonly said that no policy guidelines on alcohol consumption and ART were available. Some had heard of SANCA or similar organisations. They would also like more training to deal with substance abuse. The counsellors had some knowledge of alcohol and ARV interactions and knew that both alcohol and ARVs are metabolised in the liver and thus that alcohol impacts negatively on ARV efficacy. The counsellors contended that many patients were illiterate and that to some extent this impeded the counselling process. Expanding on particular difficulties with patients abusing alcohol, they noted that when alcohol was used as a coping mechanism: “… when they cannot accept the fact that they are living with the virus they end up drinking when they never drank before”.

Furthermore, the counsellors held that patients were dishonest about their alcohol use. In this study, there was evidence of the dual stigmas related to alcohol consumption, and HIV and AIDS. The stigma associated with alcohol consumption results in patients often not being forthcoming about the extent of their alcohol consumption. “… if you telling a patient that’s smelling of wine that ‘excuse me are you intoxicated?’ they will say ‘No, are you mad? It’s Tuesday I drank on Saturday, I just didn’t wash my clothes, but I drank on Saturday and I didn’t brush my teeth for three days’, you know things like that they will tell you…” This demonstrates that even if patients admit to drinking alcohol they are not entirely truthful and do not give accurate information concerning the volume and patterns of their alcohol use. One counsellor stated that she had difficulty dealing with some patients who were abusing alcohol as they were adept at concealing the problem and were in denial that they even had a problem.

The stigma linked to HIV-status sometimes results in the non-disclosure of a person’s HIV status. One counsellor stated that: “It is the young ones that don’t disclose to their partners because they going to lose their relationship.” The non-disclosure of HIV status to intimate partners because of the stigma associated with HIV and AIDS may result in patients not taking ARVs. The individual on ART may not want to be seen taking antiretroviral drugs: “… every time she goes into the bathroom she takes her toilet set because in the set there are ARVs, say that day you are lazy to take a bath that means that day you won’t take medication.” Non-adherence in turn has implications for optimal clinical patient management.

The counsellors were aware of the need for counselling for substance abuse. “If he goes for alcohol I can say that he needs counselling to deal with this matter”. Some patients go to Alcoholics Anonymous (AA) and “then just don’t go anymore”.

One interviewee said she did not have a right to tell her patients not to drink while others tended to have a resigned attitude to patients with such a problem. “Everybody drinks, it is part of the culture” and “you cannot tell people to stop drinking…they will continue drinking no matter what we say”. In general, they recommended reducing the amount of alcohol consumed. “I’m not saying don’t drink, seeing that you are already addicted to alcohol, just drink within limits, but what’s important is that your treatment comes first”. If a patient arrives at clinic inebriated, medication cannot be withheld. The best they can do is to provide the ARVs and ask the patient to come back sober the next day with a relative or friend to whom they have disclosed their HIV status.

### Congruence of knowledge and perception of roles among PHCWs at one specific clinic

While PHCWs’ information and knowledge differed across the clinics sampled, it also differed within clinics. This was demonstrated in the study by comparing information received from the three PHCW groups at one randomly selected clinic.

At the clinic under scrutiny, discrepancies were noted among the three cohorts of health workers in their levels of knowledge and in particular, whether there were policy guidelines on alcohol consumption and ARVs. Perceptions of competence among the healthcare providers at this clinic, in terms of providing adequate information to patients, corresponded to those observed in the study as a whole.

For example, looking at the interview responses to gauge levels of knowledge pertinent to alcohol use and ART, the counsellor seemed less informed than the doctor and the nurse. The doctor stated that he “did not know the depth of the counsellor’s knowledge.” The nurse attributed the lack of adherence to ART to: “… not enough information given to them (patients) when they were counselled.” This is a concern given the counsellor’s role in educating patients beginning to use ARVs. Furthermore, the doctor said that his job was made easier by patients already having seen the counsellor and nurse when they came to an appointment with him as he was able to build on a foundation of knowledge already laid by the other clinic staff. This assumption, however, may not be valid as nurses identified lack of sufficient information transfer in the counselling process as a reason for non-adherence to ARVs. The counsellor stated: “People even if they do start the treatment they ask, ‘Hey Sisi [counsellor] can I take a glass of wine?’ and you don’t know what to say to the patient”. The counsellor said she would like to attend workshops to answer these types of questions.

At the clinic where the interviewee responses were compared, alcohol abuse was not considered the foremost problem. All three PHCWs found it difficult to quantify the extent of the problem, saying they relied on the questions relating to alcohol use that were part of the stationery, i.e. the CAGE questions, in the patient files.

All the healthcare practitioners at the same clinic felt that they could easily identify those who came to the clinic intoxicated. For the nurses, the discernible symptoms included red eyes, irritability, the reek of alcohol or “iphuzo face,” a colloquialism for a hangover. The doctors listed symptoms such liver function test abnormalities or Dupuytren’s contracture as symptoms indicative of alcohol misuse.

Their views on the availability of policy guidelines were mixed. The doctor and counsellor claimed that there were no policy guidelines on alcohol consumption and ARVs. The nurse claimed there were very clear guidelines, but did not elaborate further. She claimed that those who abuse alcohol should not start ARVs because “of the risk of the starting medication, stopping medication and developing (drug) resistance at the end of the day”. The counsellor said that she advises patients to cut down or stop drinking alcohol when starting ARV medication. The doctor said the amount of alcohol consumed by the patient guided his advice to the patient regarding alcohol consumption and ARVs and stressed not taking ARVs and alcohol together since the alcohol inhibits absorption of the ARVs.

### Extent of the alcohol problem

The clinics in the study provided a wide range of estimates on the extent of alcohol abuse. At the clinic where the responses were compared, all three PHCWs agreed that alcohol consumption was not the foremost problem. Three other estimates emanating from the doctor, nurse, and counsellor groupings in three randomly selected clinics in the sample yielded widely differing estimates on the extent of the problem. It may be that alcohol problems are higher in some communities and it would be useful to identify the clinics serving these communities and focus on training at these clinics.

### Health service administrator interview results

The administrative officials had quite different views on the availability of guidelines on alcohol consumption and ART.

One claimed that there were specific guidelines for HIV-positive patients such as those relating to avoiding alcohol when on TB medication, but that no specific advice was given regarding alcohol consumption together with taking ARVs. The other respondent said there were no specific guidelines on alcohol consumption for HIV-positive patients in the public health sector. Both respondents, however, considered it inadvisable to mix ARVs and alcohol. The adverse effect of alcohol on the immune system and the fact that consumption negatively affects resistance to diseases and interferes with the breakdown of medications in the body was discussed. Other aspects such as the effect of alcohol consumption on liver disease were not emphasised.

One interviewee highlighted mixed messages as abstinence from alcohol consumption and drinking within normal safe limits were both endorsed. A further response was that ARVs were not withheld when a person was deemed to have a problem with alcohol consumption. The other respondent said that this would depend on the patient’s behaviour, for example, the patient’s clinic attendance.

## Discussion

Notwithstanding the PHCWS having different types of expertise and various levels of training and experience, analysis of the interviews points to inadequacies in the domain under study. The study shows a scarcity of counselling skills and the lack of clarity for PHCWs regarding guidelines on alcohol use when on ART. Other problems identified included misunderstanding of PHCWs’ recommendations and misinformation among ART clinic attendees concerning alcohol consumption and the simultaneous use of ARVs.

Research by Burman et al. [8] found that most primary care patients who misused alcohol did not receive alcohol counselling from their care providers. The PHCWs predominantly advised abstention. McCormick et al. [29] also found that most providers did not educate their primary care patients about recommended drinking limits. The study found that uneasiness on the part of the provider during alcohol-related discussions was an important barrier to evidence-based, brief alcohol counselling. The lack of knowledge and other interpersonal factors also play a role here. The findings from this study conducted in HIV and AIDS primary healthcare clinics corroborate this.

Standard drinking guidelines for HIV-positive patients and those on ART may not exist. Drinking guidelines for people on ARVs may depend on, *inter alia*, past alcohol consumption and the general state of health which includes other co-morbidities, the length of time on ART and the hepatotoxicity of certain ARVs. In terms of drinking guidelines for PLHA, Justice et al. [22] found no evidence of a “safe” level of alcohol consumption, while Conigliaro et al. [14] stated that the quantity and frequency of consumption constituting a measurable harm for HIV patients has not been determined, but would probably be lower than the amounts recommended for the general population. People with HIV have a lower tolerance for alcohol [7, 30] and hence should be informed accordingly.

In addition to the scarcity of such information for people on ART, there is the problem of misinterpreting available recommendations. For example, the doctors in this study reported that although patients are told that drinking alcohol and taking ARVS is inadvisable, the patients still jettison the ARVs and choose to drink instead.

The findings from a prospective cohort study [25] confirmed those of an earlier cross-sectional study, [24] that a substantial number of people intentionally skip or stop their medication when drinking. Multivariate analyses showed that interrupting ART when drinking alcohol occurred over and above other common correlates of non-adherence, including the frequency of alcohol consumption itself [24]. ART interruptions also occur in response to a belief, termed, interactive toxicity belief. This unfounded belief goes beyond the cognitive effects of alcohol intoxication and results in the interruption ART as a deliberate choice based on the belief that mixing alcohol with medications will lead to adverse health reactions. Some of the doctors interviewed specifically referred to interactive toxicity beliefs, although they did not use this terminology. Missing medication doses when drinking for whatever reason threatens the long-term benefits of treatment [23].

The non-disclosure of alcohol use by patients has been identified as problematic. The non-disclosure of alcohol consumption stymies the identification of problem drinkers. A study by Hahn et al. [19], a first for sub-Saharan Africa, showed that when a biomarker measurement of alcohol exposure such as a breath test is conducted together with a survey, the self-reporting of alcohol consumption improves. It may therefore be necessary to include such inexpensive and non-invasive ways to improve the self-reporting of alcohol consumption.

The South African healthcare system currently focuses on providing tertiary care services for the treatment of substance dependence where there is often a poor outcome. This focus needs to shift towards the cost-effective strategy of providing BIs early on for alcohol use disorders [35].

PHCWs at the ART clinics selected for the study do not receive adequate training on substance abuse. They generally have no knowledge of the definition of alcohol misuse or the recommended guidelines for moderate drinking. Although not particularly knowledgeable about HIV and AIDS and concomitant alcohol consumption, they are aware that an important component of treating such patients is reducing and keeping alcohol consumption to a minimum with the eventual aim of complete cessation.

At the clinic where the responses of the three PHCWs were compared, the results indicated a general lack of consensus concerning information in this domain; the information was not coherent and no clear treatment guidelines were provided. For the PHCWs there could also be different interpretations of what constitutes guidelines.

From the third tier interviews at administrative level, no comprehensive guidelines for PHCWs appeared to exist to inform patients about the negative effects of consuming alcohol at the time of, before, or subsequent to taking ARVs. The consumption of alcohol while on ART was not considered to be a priority public health concern. This is in line with what Fritz et al. [18], refer to as the “conspicuous absence” of alcohol from HIV and substance use research and programming. They suggest that the pervasiveness of alcohol use is the reason why the insidious role of alcohol consumption in the HIV epidemic has been overlooked.

### Limitations

Biomarkers were not used to validate the clinic attendees’ self-reporting of their consumption of alcohol. Furthermore, PHCWs reported that they did not employ alcohol-use screening tools to detect AUDs or less grave alcohol use problems for the clinic attendees in this study. The lack of biomarker use and alcohol-use screening tools thwarts optimal patient care in this domain. It may be that clinic attendees’ alcohol consumption is not considered to be sufficiently relevant for ART optimisation.

The Adult AIDS Clinical Trials Group, [11] an instrument for measuring levels of adherence to ART and reasons for missed doses, was not used to analyse the transcripts but could be considered for use in future research in this area focusing on adherence measurement. Overall, the findings based on interviews with PHCWs in part of the Western Cape province may not be applicable to the other eight provinces in South Africa, (or even other parts of the same province), as they have differing population compositions and administrations.

The selection of nurses and counsellors and doctors was not a random procedure. The managers may have allocated interviewees based on logistics or may have assigned more experienced personnel to be interviewed. This sampling may have resulted in a skewed PHCW sample and the findings therefore may not apply to less experienced clinic staff. Furthermore, only one doctor may have been working at the particular clinic and this clinician would be interviewed.

For the third tier of interviews, only two health service administrators were interviewed. Additional information on policy guidelines in the Western Cape metropole may have been obtained by interviewing other administrative personnel.

## Conclusions

Clear prevention and treatment guidelines should be drawn up for PHCWs and patients at ART clinics concerning alcohol consumption for patients on ARVs as well as treatment-naïve patients. Although the interconnections between alcohol use and the disease course of HIV and AIDS have not been fully delineated, guidelines regarding alcohol consumption and HIV treatment are nevertheless needed for clinic attendees. Results from this survey to assess knowledge and practices around HIV and alcohol consumption can contribute to such guidelines. These contributions include indicating where relevant information is not available. Healthcare providers could use these guidelines for their patients resulting in improved HIV treatment at primary healthcare level. Longer-term, it is anticipated that there would be buy-in from relevant stakeholders, including provincial and national health governments, clinicians, nurses and counsellors, which would hopefully influence public health initiatives impacting on the interface of ART and harmful alcohol consumption.

The study results also indicate that the service providers need additional information and training to address substance abuse in PLHA. Further training is essential for primary healthcare service providers, specifically ART counsellors in the Western Cape and explicitly on antiretroviral medication and the concomitant drinking of alcohol.

Nurses and counsellors at ART clinics need more information on both the negative direct and indirect effects of alcohol consumption for their patients. These include additional information and education around, for example, the direct negative effect of alcohol on the immune system and the indirect effects of alcohol on HIV treatment adherence and treatment outcomes [5].

### Improve levels of information

Once the level of knowledge in the target population has been determined, it is necessary to build on it, which includes educating the community at large, extending treatment networks and disseminating pertinent information. As well as nurses and counsellors at the ART clinics, HIV clubs, treatment buddies, community care workers, social workers and psychiatric nurses should receive information and training about substance abuse and ways to facilitate necessary behaviour change interventions. This information should include where to seek specialised help if necessary.

Local communities can be educated on the negative effects of alcohol on HIV and AIDS prognosis by providing health information material in the form of brochures and posters in the clinics and incorporating expanded information levels using social networks that include peer educators and HIV-clubs. The latter operate to provide medication to stable ART patients.

### Address misinformation

More information needs to be made available, specifically for clinic attendees, and misinformation needs to be addressed concerning alcohol consumption while taking antiretroviral drugs. In particular, interventions are needed to correct alcohol-related interactive toxicity misinformation and promote ARV adherence among alcohol drinkers [25]. Clinicians should educate patients to take ARVs without interruption, even when drinking. In addition, even if HIV-positive individuals do consume alcohol, ART is not denied; making this known may result in more patients receiving ARVs timeously [16].

### Treatment options

Findings from the review by Hendershot et al. [21] on alcohol use and antiretroviral adherence support the need for intervention to address alcohol in the context of highly active antiretroviral therapy. These interventions should include strategies to maximise adherence among those who are unlikely or reluctant to stop consuming alcohol.

In order to advise patients appropriately, PHCWs need knowledge of abstinence from alcohol consumption and harm reduction models [28]. Treatment options as to whether abstinence from alcohol consumption or harm reduction policies are advocated should be clarified, and indications as to when one or the other is more appropriate, specified. Furthermore, knowledge of places to refer patients identified as requiring specialised treatment for alcohol dependence or addiction should be readily available for PHCWs.

### Harm reduction

Alcohol reduction strategies need to be included in ART programmes. Instead of simply advising patients to reduce alcohol consumption, counsellors should offer practical advice on how to drink at a level that is less risky to health. This would include trying to set limits on the number of drinks, drinking beverages with low alcohol content alternating with non-alcoholic drinks, drinking slowly, and eating before or while drinking alcohol.

### Integration of services

The systematic review by Nakimuli-Mpungu et al. [32] evaluated estimates of depression, alcohol use or disorders and their association with ART adherence in sub-Saharan Africa. An important finding was that interventions to improve the mental health of HIV-positive individuals and to support ART adherence are urgently needed. In particular, to improve the integration of substance abuse services, and HIV and AIDS prevention and treatment, Parry et al. [33] stress the importance of reducing the vertical nature of HIV and AIDS and alcohol programmes. Staff require cross training at HIV and AIDS and alcohol facilities, with a focus on facilitating behaviour change, i.e. to help people change behaviour patterns that are detrimental to health.

### Part of standard care

Most (77 %) of a systematic review of 53 clinical research papers by Vagenas et al. [38] found a negative association between alcohol consumption and the HIV continuum of care. The latter represents a sequence of targets for intervention that can result in viral suppression, which benefits individuals and society. A recommendation from the study is that problematic alcohol consumption should be targeted using evidence-based behavioural and pharmacological interventions to help increase the proportion of people living with HIV achieve viral suppression to reduce HIV transmission.

A South African study by Kekwaletse and Morojele [27] on alcohol drinkers at HIV clinics, which examines the association between alcohol use and ART non-adherence points to a need for alcohol-focused behavioural interventions to improve ART adherence. The study results show that patients are amenable to clinic-based, brief, motivational interviewing and cognitive behavioural therapy-type adherence interventions, delivered by lay persons in group settings. The use of alcohol screening and brief interventions (SBI) as early interventions for problem drinkers as part of standard primary healthcare in ART clinics should be considered. Numerous studies have reported that BIs, which can take place in various settings, including primary healthcare settings, and can be implemented by a variety of trained behavioural and primary healthcare providers, are effective in reducing excessive drinking [26]. BIs consisting of up to five counselling sessions, even those lasting up to 5 min, have been found to be effective [3, 4]. Babor et al. [3] advocated that SBI training packages are necessary for the widespread dissemination of this intervention. This programme delivered in the context of a comprehensive SBI implementation programme was found to be successful in improving the providers’ knowledge, attitudes and practice of SBI for at-risk drinking.

Similar findings were reported in a study by Peltzer et al. [34] using a SBI training package developed by the World Health Organization to train primary care nurses in South Africa. To improve SBI implementation as a routine practice, the foci should be on training modalities, clinic organisation and changes in the nurse attitudes. As part of a standard care procedure, healthcare providers should routinely screen and counsel patients regarding alcohol problems to minimise HIV disease progression [13].

Furthermore, as patients in HIV clinics rarely follow referrals for outside treatment, Hasin et al. [20] suggest using smartphone technology after a BI to improve drinking outcomes in HIV-positive, alcohol dependent patients. This may offer a way of extending patient engagement with few additional demands on staff time.

In addition to making the HIV and AIDS service users and providers more attentive to potential alcohol abuse problems, it is anticipated that this study will generate public and health policy makers’ awareness concerning the detrimental health effects of excessive alcohol consumption for treatment-naïve HIV patients and those taking ARVs.

## Abbreviations

AA: Alcoholics Anonymous
AACTG: Adult AIDS Clinical Trials Group
AIDS: Acquired immunodeficiency syndrome
APA: American Psychiatric Association
ART: Antiretroviral treatment
ARV: Antiretroviral
ATODRU: Alcohol, Tobacco and Other Drug Research Unit
AUD: Alcohol use disorder
AUDIT: Alcohol Use Disorders Identification Test
BI: Brief intervention
CAGE: Cutting down, Annoyance by criticism, Guilty feeling and Eye-openers
CDC: Centers for Disease Control and Prevention
DSM: Diagnostic and Statistical Manual of Mental Disorders
HIV: Human immunodeficiency virus
ICD: International Classification of Disease
PEPFAR: President’s Emergency Plan for AIDS Relief
PHCW: Primary healthcare worker
PI: Principal Investigator
PLHA: People living with HIV and AIDS
SANCA: South African National Council on Alcoholism and Drug Dependence
SBI: Screening and brief interventions
WHO: World Health Organization

## Footnotes

1 The CAGE questionnaire, the name being an acronym of its four questions, is a widely used method of screening for alcoholism.

2 Binge drinking can be defined as five or more drinks in a row for males, or four or more drinks in a row for females over a few hours, or drinking large amounts over several days as an established drinking pattern. This corresponds to 60 g of pure alcohol [34]. The definition of a standard drink differs from country to country; in South Africa, it is generally accepted that a drink containing 12 g of alcohol would be regarded as standard.
